# Supplementary material for: Deletion of the moeA gene in Flavobacterium IR1 drives structural color shift from green to blue and alters polysaccharide metabolism
Source: eLife. 2026 Jan 2;14:RP105029. doi: 10.7554/eLife.105029 (PMC12758843; doi:10.7554/eLife.105029)
Supplement: Supplementary file 1. — a. Bacterial strains used in this study. b. Plasmids used in this study. c. Oligonucleotides used in this study. d. The most downregulated intracellular proteins in the ΔmoeA mutant, and proteins mentioned in the main text and in Figure 8. * Gene name or protein with the designation that gives the most information. “-” means no gene name; hyp: hypothetical; TP: transporters. e. The 5 most upregulated intracellular proteins in the ΔmoeA mutant, and proteins mentioned in the main text and in Figure 8. * Gene name or protein with the designation that gives the most information. “-” means no gene name; hyp: hypothetical; GT-2: glycosyltransferase family 2; GHX: glycosyl hydrolase family X; TRX: thioredoxin domain-containing protein; TBDR: TonB-dependent receptor; OMP: outer membrane protein; CLB: colibactin biosynthesis ABH: alpha/beta hydrolase. f. The most 5 downregulated extracellular proteins in the ΔmoeA mutant, and proteins mentioned in the main text and in Figure 8. * Gene name or protein with the designation that gives the most information. “-” means no gene name; hyp: hypothetical. ** SP: signal peptide; NC: non-classical; -: not secreted. g. The 5 most upregulated extracellular proteins in the ΔmoeA mutant, and proteins mentioned in the main text and in Figure 8. * Gene name or protein with the designation that gives the most information. ABH: alpha/beta hydrolase; SP: secreted protein; CF: cell surface. ** SP: signal peptide; NC: non-classical; -: not secreted. [file elife-105029-supp1.docx]

**SUPPLEMENTARY FILE**

**a.** Bacterial strains used in this study.

| **Bacterial strains** | **Relevant characteristics** | **Source** |
| --- | --- | --- |
| *Escherichia coli* DH5α | Strain used for general cloning | NEB |
| *Flavobacterium* IR1 | Wild-type (WT) | Johansen et al., 2018 |
| *Flavobacterium* IR1 Δ*moe*A | IR1 *moe*A knock-out (KO) via SIBR | This study |

**b.** Plasmids used in this study.

| **Plasmid name** | **Description** | **Source** |
| --- | --- | --- |
| pSIBR048 | *omp*Ap-Int3 FnCas12a-mapt, Hup-NT spacer-*omp*At, Spec^R^ (*E. coli*), Erm^R^ (IR1) | (Patinios et al., 2021) |
| pMoeA_NT | pSIBR048 containing homologous arms of *moe*A | This study |
| pMoeA_S1 | pSIBR048 containing homologous arms of *moe*A and targeting spacer 1 for *moe*A | This study |

**c.** Oligonucleotides used in this study.

| **Oligo ID** | **Sequence** | **Description** |
| --- | --- | --- |
| cHA fwd | TCCTCCTTAGCTCAGTTGGTTAG | To check the introduction of the homologous arms into the plasmid, forward |
| cHA rev | CAGGAAACAGCTATGACCATG | To check the introduction of the homologous arms into the plasmid, reverse |
| cSp fwd | CAGGAAACAGCTATGACCATG | To check the introduction of the spacer into the plasmid, forward |
| cSp rev | CCAACACTTGCAAGGAACGG | To check the introduction of the spacer into the plasmid, reverse |
| *moe*A US fwd | GGCCTCGAGATCTCCATGGATATTTCCCAAGATGAATTTG | Upstream homologous arm for *moe*A, forward |
| *moe*A US rev | GCTATTTTATAAGGTAAGCA | Upstream homologous arm for *moe*A, reverse |
| *moe*A DS fwd | TGCTTACCTTATAAAATAGCAGCAGTGTGTAAATTTAAAC | Downstream homologous arm for *moe*A, forward |
| *moe*A DS rev | CCTGCAATAAATCCTGCAGT | Downstream homologous arm for *moe*A, reverse |
| cHA inner *moe*A | TAAAGATGCAGGCGTTTACG | To check the insertion of the homologous arms of *moe*A |
| *moe*A S1 fwd | TGGTCTCTTAGACATTATTGCGCAAAATAGTACATCTATGAGACCT | *moe*A spacer insertion 1, forward |
| *moe*A S1 rev | AGGTCTCATAGATGTACTATTTTGCGCAATAATGTCTAAGAGACCA | *moe*A spacer insertion 1, reverse |
| cFwd *moe*A | GCTGTATAGGATGTAAAGCC | To check the deletion of the *moe*A gene in the genome, forward |
| cRev *moe*A | TAAAGATGCAGGCGTTTACG | To check the deletion of the *moe*A gene in the genome, reverse |

**d.** The most downregulated intracellular proteins in the Δ*moe*A mutant, and proteins mentioned in the main text and in Figure 8.

| **Role** | **ID protein** **(GenBank)** | **Protein name** | **Gene name*** | **Fold**  **change** |
| --- | --- | --- | --- | --- |
| Molybdenum cofactor (MoCo) synthesis | PAM94797 | Molybdopterin molybdenum transferase | *moe*A | -6.64 |
| Purine catabolism | PAM91437 | 2Fe-2S ferredoxin | *yag*T | -3.94 |
|  | PAM91438 | FAD-binding molybdopterin dehydrogenase | *yag*S | -1.82 |
|  | PAM91439 | Aldehyde oxygenase | *yag*R | -3.20 |
| Fatty acid biosynthesis | PAM91878 | DUF983 domain-containing protein | *hyp*1 | -1.18 |
|  | PAM91879 | [acyl-carrier-protein] S-malonyltransferase | *fab*D | -2.02 |
| Phospholipid transformation | PAM93675 | ABC transporter ATP-binding protein | *mla*F | -6.64 |
|  | PAM93677 | Membrane assembly protein | *asm*A | -6.64 |
| Nitrogen assimilation | PAM94801 | Nitrite reductase | *-* | -1.15 |
| Translation | PAM92040 | Ribosomal protein L27 | *rpm*A | -1.57 |
|  | PAM92136 | Ribosomal protein S15 | *rps*O | -1.54 |
|  | PAM94293 | Ribosomal protein L16 | *rpl*P | -1.35 |
| RNA processing | PAM93099 | tRNA(guanosine(37)-N1)-methyltransferase | *trm*D | -1.38 |
|  | PAM93366 | Proline—tRNA ligase | *pro*S | -1.25 |
|  | PAM96417 | tRNA(adenosine(37)-N6)-threonylcarbamoyltransferase complex ATPase subunit type 1 | *tsa*E | -2.70 |
| DNA transcription | PAM92509 | RNA polymerase sigma-54 factor | *rpo*N | -1.23 |
|  | PAM93777 | RNA polymerase sigma-19 factor | *fec*I | -3.52 |
| Amino acid biosynthesis | PAM91434 | Alanine dehydrogenase | *xdh*C | -6.64 |
|  | PAM94076 | Type II 3-dehydroquinate dehydratase | *aro*Q | -1.41 |
|  | PAM95008 | Acetylornithine deacetylase | *arg*E | -1.33 |
|  | PAM95600 | Glutamine synthetase | *gln*A | -1.89 |
| Antimicrobial resistance | PAM93287 | Transporter | TP | -1.90 |
|  | PAM93288 | Hydrophobe/amphiphite efflux-1 family RND transporter | *mdt*F | -1.17 |
|  | PAM93289 | Efflux transporter periplasmic adaptor unit | *mdt*E | -2.52 |
| Unknown | PAM92103 | Hypothetical protein | *hyp*A | -6.64 |
|  | PAM93709 | Hypothetical protein | - | -6.64 |

* Gene name or protein with the designation that gives the most information. “-” means no gene name; *hyp*: hypothetical; TP: transporters.

**e.** The 5 most upregulated intracellular proteins in the Δ*moe*A mutant, and proteins mentioned in the main text and in Figure 8.

| **Role** | **ID protein** **(GenBank)** | **Protein name** | **Gene name*** | **Fold change** |
| --- | --- | --- | --- | --- |
| Molybdenum cofactor (MoCo) synthesis | PAM94790 | GTP 3’,8-cyclase | *moa*A | 2.18 |
|  | PAM94791 | Cyclic pyranopterin monophosphate synthase 2 | *moa*C2 | 2.15 |
|  | PAM94796 | Molybdenum cofactor guanylyl transferase | *mob*A | 3.84 |
| Nitrogen assimilation | PAM94787 | NAD(P)H-nitrite reductase | *nir*B | 1.05 |
| Cell wall synthesis | PAM94230 | Hypothetical protein | *hyp*2 | 1.23 |
|  | PAM94231 | Hypothetical protein | GT-2a | 2.32 |
|  | PAM94234 | Hypothetical protein | GT-2b | 1.92 |
|  | PAM94238 | ABC transporter ATP-binding protein | *tag*H | 1.24 |
| Respiratory electron transport | PAM91938 | Cytochrome oxidase subunit III | *cyo*B | 1.00 |
|  | PAM91940 | Protoheme IX farnesyltransferase | *cyo*E | 2.32 |
| Carbohydrate metabolism | PAM95090 | Xylosidase | GH43 | 1.03 |
|  | PAM95092 | Beta-glucosidase | *bgl*X | 1.11 |
|  | PAM95094 | Nutrient uptake outer membrane protein | *sus*D1 | 1.06 |
|  | PAM95388 | Laminarase | GH16 | 1.07 |
|  | PAM95389 | Hypothetical protein | *hyp*3 | 1.52 |
| Stress response | PAM94360 | Chalcone isomerase | - | 4.14 |
|  | PAM94935 | DUF6734 family protein | TRX1 | 3.39 |
|  | PAM94936 | Hypothetical protein | TRX2 | 1.84 |
|  | PAM94937 | Hypothetical protein | *hyp*4 | 1.62 |
| Signal transduction | PAM95501 | Response regulator | - | 3.59 |
| RNA processing | PAM93105 | 23S rRNA (adenine(1618)-N(6))- methyltransferase | *rlm*F | 5.47 |
| Regulation of DNA transcription | PAM92290 | TetR family transcriptional regulator | *acr*R | 1.38 |
|  | PAM93723 | Transcriptional regulator | *omp*R | 2.41 |
|  | PAM94944 | Transcriptional regulator | *hip*B | 3.84 |
| Proteolysis | PAM96640 | Aminopeptidase | AP1 | 4.04 |
| Non-ribosomal peptide synthesis | PAM96235 | Hypothetical protein | CLB | 1.78 |
|  | PAM96237 | Hypothetical protein | CLB1 | 2.83 |
|  | PAM96238 | Hypothetical protein | *clb*I | 2.21 |
|  | PAM96239 | Hypothetical protein | *ent*F | 3.03 |
|  | PAM96240 | Hypothetical protein | *clb*B | 2.11 |
|  | PAM96241 | Hypothetical protein | TBDR | 1.60 |
|  | PAM96242 | Hypothetical protein | CLB2 | 2.42 |
|  | PAM96243 | Hypothetical protein | OMP | 1.14 |
|  | PAM96245 | Thioesterase | *clb*Q | 1.77 |
|  | PAM96247 | Non-ribosomal peptide synthetase | CLB3 | 1.40 |
|  | PAM96501 | 4-phosphopantetheinyl transferase | *clb*A | 1.83 |
|  | PAM96502 | Alpha/beta hydrolase | ABH1 | 2.61 |
| Unknown | PAM96476 | Hypothetical protein | *hyp*B | 4.92 |

* Gene name or protein with the designation that gives the most information. “-” means no gene name; *hyp*: hypothetical; GT-2:glycosyltransferase family 2; GHX: glycosyl hydrolase family X; TRX: thioredoxin domain-containing protein; TBDR: TonB-dependent receptor; OMP: outer membrane protein; CLB: colibactin biosynthesis ABH: alpha/beta hydrolase.

**f.** The most 5 downregulated extracellular proteins in the Δ*moe*A mutant, and proteins mentioned in the main text and in Figure 8.

| **Role** | **ID protein**  **(GenBank)** | **Protein name** | **Gene**  **name*** | **Secretion**  **pathway**** | **Fold**  **change** |
| --- | --- | --- | --- | --- | --- |
| Protein modification | PAM93429 | Peptidylprolyl isomerase | *fkp*A | SP | -3.00 |
|  | PAM93873 | Glutamine cyclotransferase | - | SP | -2.18 |
| Carbohydrate metabolism | PAM91916 | Galactose oxidase | *-* | SP | -1.48 |
|  | PAM91646 | Nutrient uptake outer membrane protein | *sus*C | SP | -1.03 |
| Stress response | PAM94872 | Superoxide dismutase | *sod*C | SP | -1.51 |
| Cell division | PAM92714 | Murein hydrolase activator | *env*C | SP | -3.51 |
| Antibiotic resistance | PAM96491 | Serine hydrolase | *-* | SP | -2.40 |
|  | PAM91787 | Serine hydrolase | - | SP | -1.71 |
| Lipopolysaccharide assembly | PAM94009 | Hypothetical protein | *hyp*G | NC | -3.13 |
|  | PAM95883 | Hypothetical protein | - | SP | -2.19 |
| Electron transport | PAM91962 | Cytochrome C | *cta*D | SP | -1.13 |
|  | PAM95417 | Azurin | *azu* | SP | -2.82 |
| Motility | PAM91688 | Flagellin biosynthesis protein | *flg*D | SP | -4.72 |
|  | PAM91755 | Gliding motility protein | *gld*L | NC | -2.22 |
| Transport | PAM92093 | TonB-dependent receptor | *fep*A | SP | -1.98 |
|  | PAM93287 | RND transporter | *tol*C | SP | -1.95 |
| Unknown | PAM92477 | Hypothetical protein | *hyp*10 | SP | -1.93 |
|  | PAM92479 | Hypothetical protein | *hyp*11 | SP | -1.32 |
|  | PAM96055 | Hypothetical protein | *-* | SP | -2.63 |

* Gene name or protein with the designation that gives the most information. “-” means no gene name; *hyp*: hypothetical.

** SP: signal peptide; NC: non-classical; -: not secreted.

**g.** The 5 most upregulated extracellular proteins in the Δ*moeA* mutant, and proteins mentioned in the main text and in Figure 8.

| **Role** | **ID protein**  **(GenBank)** | **Protein name** | **Gene**  **name*** | **Secretion**  **pathway**** | **Fold**  **change** |
| --- | --- | --- | --- | --- | --- |
| Transport | PAM94786 | Hypothetical protein | - | SP | 2.28 |
|  | PAM96649 | Hypothetical protein | - | SP | 2.41 |
| Proteolysis | PAM93900 | Zinc metalloprotease | - | SP | 2.29 |
|  | PAM95531 | Hypothetical protein | - | SP | 2.22 |
| Carbohydrate metabolism | PAM93863 | Glycoside hydrolase family 18 | GH18 | SP | 2.42 |
|  | PAM94980 | TonB-dependent receptor | TBDR | SP | 1.39 |
|  | PAM95091 | Glycerophosphodiester phosphodiesterase | GDP | SP | 1.49 |
|  | PAM95092 | Beta-glucosidase | *bgl*X | SP | 1.78 |
|  | PAM95094 | Nutrient uptake outer membrane protein | *sus*D1 | SP | 1.10 |
|  | PAM95095 | TonB-linked outer membrane protein | *sus*C1 | SP | 1.13 |
|  | PAM95273 | Pectate lyase | *pel*B | SP | 1.41 |
|  | PAM95448 | TonB-linked outer membrane protein | *sus*C2 | SP | 1.12 |
|  | PAM95449 | Nutrient uptake outer membrane protein | *sus*D2 | SP | 1.66 |
|  | PAM95764 | Alpha/beta hydrolase | ABH2 | - | 1.10 |
| Fatty acid biosynthesis | PAM92474 | Acetyl-CoA carboxylase, biotin carboxyl carrier protein | *acc*B | NC | 2.56 |
|  | PAM92475 | Acetyl-CoA carboxylase biotin carboxylase subunit | *acc*C | - | 1.20 |
| Cell division | PAM92348 | Hypothetical protein | *hyp*F | SP | 2.76 |
| Unknown | PAM91530 | Secretion protein | SP | SP | 1.55 |
|  | PAM91622 | Cell surface protein | CF | SP | 1.09 |
|  | PAM92266 | Hypothetical protein | *hyp*E | NC | 3.11 |
|  | PAM92573 | Hypothetical protein | *hyp*D | SP | 4.05 |
|  | PAM93697 | Hypothetical protein | *hyp5* | SP | 1.43 |
|  | PAM93700 | Hypothetical protein | *hyp*6 | NC | 1.97 |
|  | PAM93702 | Hypothetical protein | *hyp*7 | SP | 1.50 |
|  | PAM95724 | Hypothetical protein | *hyp*8 | SP | 1.65 |
|  | PAM95725 | Hypothetical protein | *hyp*9 | SP | 1.18 |

* Gene name or protein with the designation that gives the most information. ABH: alpha/beta hydrolase; SP: secreted protein; CF: cell surface.

** SP: signal peptide; NC: non-classical; -: not secreted.
